# SILAC-Based Quantification of TGFBR2-Regulated Protein Expression in Extracellular Vesicles of Microsatellite Unstable Colorectal Cancers

**DOI:** 10.3390/ijms20174162

**Published:** 2019-08-26

**Authors:** Fabia Fricke, Malwina Michalak, Uwe Warnken, Ingrid Hausser, Martina Schnölzer, Jürgen Kopitz, Johannes Gebert

**Affiliations:** 1 Department Applied Tumor Biology, Institute of Pathology, Heidelberg University Hospital, Im Neuenheimer Feld 224, 69120 Heidelberg, Germany; 2 Clinical Cooperation Unit Applied Tumor Biology, German Cancer Research Center (DKFZ), Im Neuenheimer Feld 224, 69120 Heidelberg, Germany; 3 Functional Proteome Analysis, German Cancer Research Center (DKFZ), Im Neuenheimer Feld 581, 69120 Heidelberg, Germany; 4 EM-Lab, Institute of Pathology, Im Neuenheimer Feld 224, Heidelberg University Hospital, 69120 Heidelberg, Germany

**Keywords:** extracellular vesicles, exosomes, proteomics, stable isotope labeling with amino acids in cell culture (SILAC), TGFBR2, microsatellite instability, DNA mismatch repair, colorectal cancer

## Abstract

Microsatellite unstable (MSI) colorectal cancers (CRCs) are characterized by mutational inactivation of *Transforming Growth Factor Beta Receptor Type 2* (*TGFBR2*). TGFBR2-deficient CRCs present altered target gene and protein expression. Such cellular alterations modulate the content of CRC-derived extracellular vesicles (EVs). EVs function as couriers of proteins, nucleic acids, and lipids in intercellular communication. At a qualitative level, we have previously shown that TGFBR2 deficiency causes overall alterations in the EV protein content. To deepen the basic understanding of altered protein dynamics, this work aimed to determine TGFBR2-dependent EV protein signatures in a quantitative manner. Using a stable isotope labeling with amino acids in cell culture (SILAC) approach for mass spectrometry-based quantification, 48 TGFBR2-regulated proteins were identified in MSI CRC-derived EVs. Overall, TGFBR2 deficiency caused upregulation of several EV proteins related to the extracellular matrix and nucleosome as well as downregulation of proteasome-associated proteins. The present study emphasizes the general overlap of proteins between EVs and their parental CRC cells but also highlights the impact of TGFBR2 deficiency on EV protein composition. From a clinical perspective, TGFBR2-regulated quantitative differences of protein expression in EVs might nominate novel biomarkers for liquid biopsy-based MSI typing in the future.

## 1. Introduction

Microsatellite unstable (MSI) colorectal cancers (CRCs) are caused by defects of the DNA mismatch repair system [1]. The MSI phenotype is present in about 15% of CRCs and is characterized by distinct molecular and clinicopathological features [2,3], which allow precise discrimination from other CRC subtypes. From a clinical perspective, MSI tumors often develop in the proximal colon with inflammatory patterns and invasion into surrounding tissues [2]. Moreover, MSI tumors have a lower propensity to form distant metastases [4,5], and thus often have a better prognosis when compared to microsatellite stable counterparts [6]. In general, MSI tumors show altered chemo-responsiveness emphasizing the importance of alternative therapeutic strategies [7]. Recently, MSI CRCs gained increasing attention due to promising clinical responsiveness to immune checkpoint therapies [8,9], which are associated with the molecular mechanisms underlying MSI tumorigenesis. One specific molecular feature of MSI tumors is a high mutational load that results from the accumulation of somatic insertion/deletion mutations in repetitive DNA stretches (i.e., microsatellites) spread over the whole genome. While numerous microsatellite-harboring genes were identified and found to be frequently affected by irrelevant passenger mutations, frameshift mutations in only a limited number of these genes are potential MSI tumor drivers [10,11,12]. About 90% of MSI colorectal tumors exhibit inactivating frameshift mutations in a polyadenine (A10) tract in exon 3 of *Transforming Growth Factor Beta Receptor Type 2* (*TGFBR2*) gene [13,14]. TGFBR2 deficiency in colon epithelial cells is considered to drive MSI tumor progression by abrogating downstream Transforming Growth Factor-beta (TGF-β) signaling [13,15]. Missense mutations affecting *TGFBR2* were also identified in 15% of microsatellite stable colorectal tumors [16] and in several other tumor types, including breast [17], prostate [18] and renal cell carcinoma [19]. TGFBR2 is a serine/threonine kinase and the primary ligand-binding receptor. After binding of the ligand TGF-ß1, TGFBR2 forms a heterodimer complex with TGFBR1, causing conformational changes and signal propagation via canonical (SMAD-dependent) and non-canonical (SMAD-independent) routes. Upon translocation into the nucleus, activated SMAD complexes can interact with various transcription factors to control target gene expression [20,21]. Downstream targets of TGFBR2-mediated signal transduction were identified at the transcriptomic and proteomic level of CRC cells and altered target gene and protein expression has been linked to TGFBR2 deficiency [21,22,23].

Such altered cellular expression patterns can modulate the molecular cargo of MSI tumor cell—derived extracellular vesicles (EVs). EVs are a heterogeneous population of membrane-enclosed particles that can be classified into different EV subtypes according to their biogenesis and sizes. The two most prominent subclasses are exosomes (30 to 150 nm) originating from the endosomal pathway and microvesicles (100 to 1000 nm) that are plasma membrane-derived [24,25]. Since exosomes, microvesicles, and other subtypes display overlapping physical and biological properties; current EV isolation methods cannot efficiently isolate individual EV subtypes and a clear consensus allowing discrimination between individual EV subpopulations has not yet been established [24,26]. Apart from difficulties of their classification, shuttling EVs have emerged as an intercellular messaging system used by almost all cells, including CRC cells [27]. The EV-transferred information is encoded by EV cargo molecules, which can elicit a biological response in recipient cells [28]. The EV cargo covers an assortment of membrane and cytosolic components (proteins, nucleic acids, lipids, metabolites) originating from the parental cell [29]. To some extent, the EV cargo reflects the biochemical makeup of parental cells making EVs interesting targets for biomarker discovery and liquid biopsy [30]. In previous work, we have shown that the recurrent MSI tumor driver mutation affecting *TGFBR2* can modulate the cargo of MSI tumor cell-derived EVs at a qualitative level [31]. In order to gain a deeper and comprehensive understanding about EV cargo changes caused by TGFBR2 deficiency, the present work aimed to quantify protein differences between EVs derived from TGFBR2-deficient (dT, −dox) and TGFBR2-proficient (pT, +dox) HCT116 cells genetically modified to confer doxycycline (dox)-inducible *TGFBR2* expression in an isogenic background. Stable isotope labeling with amino acids in cell culture (SILAC) combined with high-resolution mass spectrometry identified 48 EV proteins that turned out to be regulated in a TGFBR2-dependent manner.

## 2. Results 

### 2.1. Isolation and Identification of EVs Secreted by TGFBR2-Deficient and -Proficient MSI Tumor Cells

**Figure 1:**
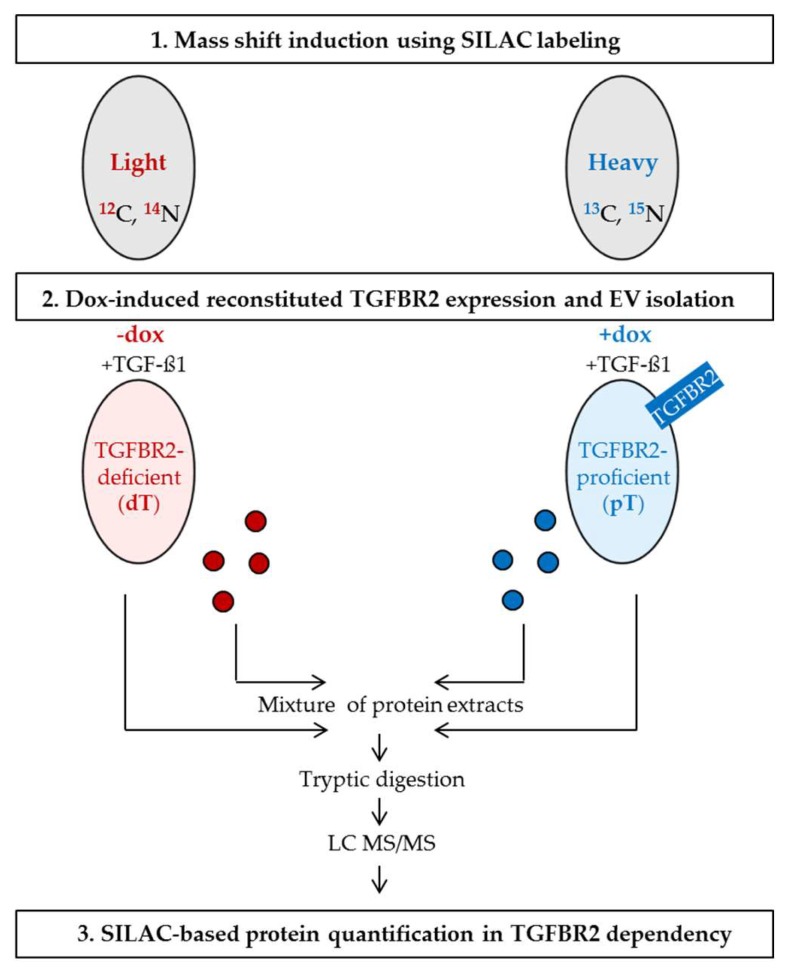
Transforming Growth Factor Beta Receptor Type 2 (TGFBR2)-dependent protein quantification using stable isotope labeling with amino acids in cell culture (SILAC). HCT116-TGFBR2 cells were labeled with light (*red*) or heavy (*blue*) amino acids. The mass shift induction was performed over a period of 14 days. Heavy-labeled cells were treated with dox to induce reconstituted expression of TGFBR2 (heavy state: TGFBR2-proficient, pT). Light-labeled cells were cultivated in the absence of dox and remained TGFBR2-deficient (light state: TGFBR2-deficient, dT). The SILAC experiment was performed in triplicate. Extracellular vesicles (EVs) were isolated and characterized from both conditions. Protein extracts from EVs and their parental cells were mixed separately and prepared for high-resolution mass spectrometry analysis. “Light-to-heavy” (dT/pT) protein ratios were calculated in order to identify proteins regulated in a TGFBR2-dependent manner.

In order to quantify the EV protein composition in a TGFBR2-dependent manner, we used our previously established human MSI cell line HCT116-TGFBR2 as a model system [32]. These cells exhibit the MSI phenotype and were genetically modified to confer dox-inducible expression of a single copy *TGFBR2* transgene. In the absence of dox, these cells are TGFBR2-deficient (−dox, dT), which mimics the condition of most primary MSI tumors that have lost receptor expression during tumor progression. In contrast, in the presence of dox, HCT116-TGFBR2 cells show reconstituted *TGFBR2* expression (+dox, pT), which allows the identification of complex molecular changes in an isogenic background. In order to prove the functionality of this MSI model system, we examined the induction of dox-mediated TGFBR2 expression and activation of downstream signaling by Western blot analysis (Supplementary Figure S1). In the absence of dox and in the presence of the ligand TGF-ß1 (−dox, +TGF-ß1; dT), these cells lacked TGFBR2 and phosphorylated SMAD2 (pSmad2) expression and thus were unable to activate downstream canonical signaling. However, upon exposure to dox and the ligand (+dox, +TGF-ß1; pT), these cells showed reconstituted TGFBR2 expression and functional downstream signaling as demonstrated by activation of pSMAD2. After verifying our model system, SILAC conditions were established for HCT116-TGFBR2 cells allowing EV isolation from three biological replicates of heavy-labeled pT-cells and light-labeled dT-cells (Figure 1). The identification of isolated EVs was performed in compliance with the minimal information for studies of extracellular vesicles (MISEV) guidelines [26] using three different approaches. 

**Figure 2:**
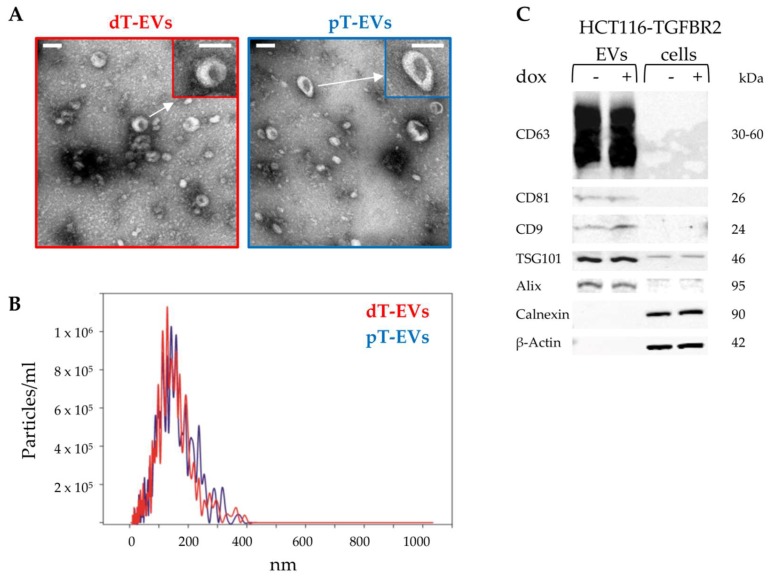
Identification of EVs. (**A**) Transmission electron microscopy (TEM) pictures visualized the morphology of isolated EVs. Scale bar: 100 nm. (**B**) Nanoparticle tracking analysis (NTA) revealed the size distribution and particle concentration of isolated EVs. (**C**) Western blot analysis confirmed EV-specific and cell-specific protein marker expression. Protein sizes are indicated.

First, transmission electron microscopy was used to visualize the sizes and structures of prepared EVs. As indicated in Figure 2A, EVs were detected as a heterogeneous population of small (<200 nm) particles showing round and cup-shaped morphologies.

Next, nanoparticle tracking analysis (NTA) confirmed the observed size distributions (Figure 2B). Median sizes of 126.3 nm (mean 135 ± 61.1 nm) 125.0 nm (mean 132.6 ± 63.3 nm) were observed for dT-EVs and pT-EVs, respectively. Particle concentrations were calculated resulting in 6.6 × 10^10^ (dT-EVs) and 6.7 × 10^10^ (pT-EVs) particles per mL. 

Finally, expression of EV-specific (CD63, CD81, CD9, TSG101, Alix) and cell-specific marker proteins (Calnexin, ß-Actin) was analyzed by Western blot analysis (Figure 2C). Membrane-associated tetraspanins CD63, CD81, and CD9 were exclusively detected in EV protein extracts but not in protein lysates of corresponding parental cells. The cytosolic proteins TSG101 and Alix were found to be enriched in protein lysates of EVs compared to their parental cells. Calnexin and cytoskeletal ß-Actin were identified in cell lysates but not in EV protein extracts, suggesting efficient EV separation from cells and cellular debris. Altogether, these results demonstrate the successful enrichment of EVs secreted from MSI tumor cells only differing in their TGFBR2 expression status. Herein, no effect of TGFBR2 deficiency on EV sizes, concentrations and protein marker expression was observed. These comprehensively characterized vesicles were used for downstream proteomic analysis.

### 2.2. SILAC-Based Quantification of EV Proteins Regulated in a TGFBR2-Dependent Manner

**Figure 3:**
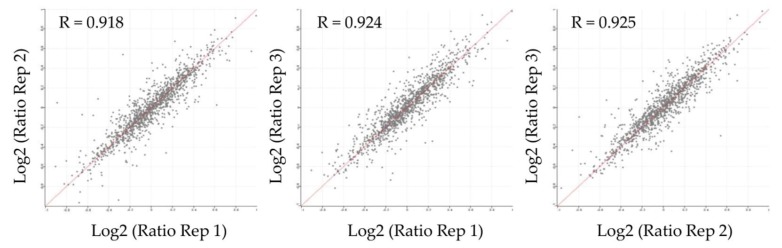
Correlation analysis of protein ratios between biological replicates of EVs. Log2-transformed protein ratios (dT/pT) of each biological replicate were plotted against each other and Pearson′s correlation coefficients (R) were calculated.

**Figure 4:**
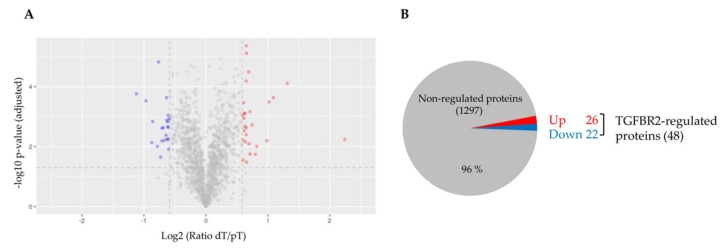
Analysis of TGFBR2-regulated proteins in EVs. (**A**) Volcano plot shows non-regulated proteins (*grey*) and significantly up- (*red*) and downregulated (*blue*) proteins of EVs derived from TGFBR2-deficient cells. *X* axis represents log_2_-transformed fold change values. *Y* axis shows the −log_10_ *p*-value adjusted for multiple comparisons; (**B**) Pie chart specifies non-regulated EV proteins (*grey*) and EV proteins up- (*red*) and downregulated (*blue*) by cellular TGFBR2 deficiency.

For accurate SILAC-based quantification of proteins, it is of utmost importance to ensure efficient incorporation of labeled amino acids. Therefore, we first analyzed the mass shift induction in EV protein lysates. Metabolic labeling of parental HCT116-TGFBR2 cells resulted in efficient (~98%) incorporation of heavy-labeled amino acids in proteins extracted from EVs (Supplementary Figure S2). Next, peptide mixtures obtained from three biological replicates of dT- and pT-EVs were prepared and analyzed by high-resolution mass spectrometry. In total, 1345 EV proteins were identified (unique peptides ≥2) and quantified in at least two out of three biological EV replicates. The quantified EV proteome encompassed 80 proteins of the top 100 EV proteins that have been identified in previous studies [33], thereby confirming the validity of our EV enrichment strategy. In order to detect protein candidates differentially expressed between dT- and pT-EVs, “light-to-heavy” (dT/pT) protein ratios were calculated for each replicate and the reproducibility was assessed. Pearson’s correlation coefficients (R) revealed a high degree of reproducibility (R ≥ 0.918) between all three replicates (Figure 3) allowing quantification of TGFBR2-dependent EV protein profiles. Although the majority (1297/1345) of EV proteins was not altered by the TGFBR2 expression status, a subset of 48/1345 EV proteins was found to be significantly (*p* < 0.05) regulated in a TGFBR2-dependent manner (Figure 4A). In particular, 26/48 EV proteins turned out to be upregulated and 22/48 EV candidates were found to be downregulated in dT-EVs (Figure 4B). Up- and downregulated EV proteins are listed in Table 1 and Table 2, respectively. Protein ratios of downregulated candidates ranged from 0.46- to 0.66-fold. Interestingly, most of them were related to the proteasome (PSMB1, PSMB2, PSMB3, PSMB5, PSMA1, PSMA2, PSMA3, PSMA5, SAFB). In contrast, upregulated candidates encompassed EV proteins whose expression levels were increased 1.50- to 4.73-fold. The majority of these were associated with the organization of extracellular matrix (FN1, FBLN1, COL6A1, COL6A2, THBS1), or the nucleosome (HIST1H4A, HIST1H2BL, HIST2H2BE, H2AFV, H2AFY). 

**Figure 5:**
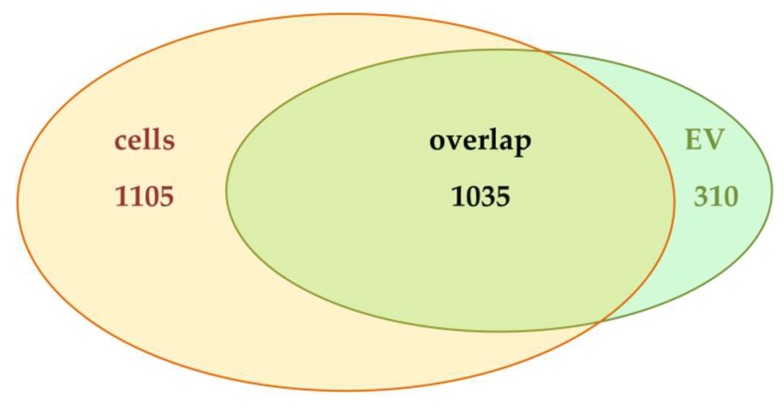
Venn diagram analysis of proteins identified in HCT116-TGFBR2 cells (*yellow*) and EVs (*green*) derived thereof. Numbers of proteins per intersection are given.

Given the fact that 48/1345 EV proteins turned out to be regulated in a TGFBR2-dependent manner, we sought to determine whether this regulation might reflect changes in the proteome of parental cells or might be found exclusively at the vesicular level. Therefore, peptide mixtures of corresponding parental dT- and pT-cells were prepared and analyzed. In cellular protein extracts, a total of 2140 proteins was identified and quantified in at least two out of three biological replicates. Among these, 1035 proteins were previously found to be also present in EVs (Figure 5). This finding implies that 48% (1035/2140) of cellular proteins appeared to be sorted into EVs, which highlights the general overlap of proteins between MSI CRC cells and their secreted EVs. In this shared subset, we cannot determine any quantitative differences in protein expression between MSI cells and their EVs, because of differences in their proteome complexity and the lack of an appropriate normalization strategy. Apart from this shared subset, EV-specific (310/1345) as well as cell-specific protein subsets (1105/2140) were identified.

In order to determine the impact of TGFBR2 deficiency on the cellular proteome, protein ratios (dT/pT) were calculated and compared to ratios of corresponding EVs. TGFBR2 expression status caused changes in relative EV protein abundance compared to parental cells as shown in the histogram (Supplementary Figure S3). At the cellular level, 14/2140 proteins were regulated in a TGFBR2-dependent manner. Among them, only 3/14 candidates showed statistical significance (Supplementary Table S1).

In summary, these results demonstrate that the majority of TGFBR2-regulated proteins was detected in MSI-derived EVs, but not in parental cells, which suggests that the observed alterations are not simply a consequence of changes in the cellular proteome but rather appear to be EV-specific.

### 2.3. Validation of TGFBR2-Regulated Protein Expression in EVs

**Figure 6:**
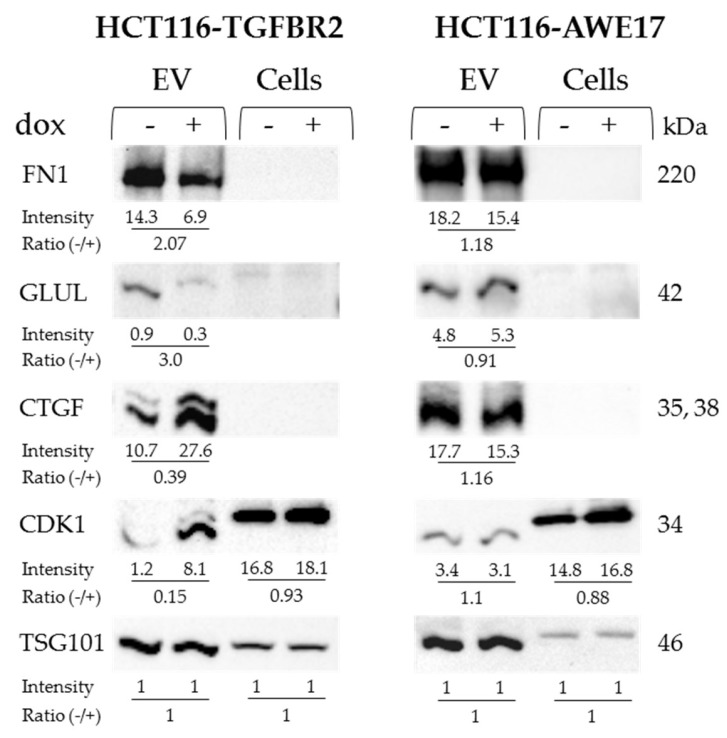
Validation of TGFBR2-regulated EV proteins. Representative TGFBR2-regulated EV proteins (FN1, GLUL, CTGF, CDK1) were analyzed on Western blots in at least two biological replicates. The non-regulated EV marker protein TSG101 was used as a loading control. EVs derived from HCT116-AWE17 cells served as a control for dox-related effects. Band intensities of proteins and calculated ratios are shown below each lane and were normalized to TSG101 set to 1. Protein sizes are indicated.

To obtain independent evidence for TGFBR2-regulated proteins identified by mass spectrometry, Western blot analysis was performed on protein lysates of dT- and pT-EVs and parental HCT116-TGFBR2 cells. The two candidates connective tissue growth factor (CTGF) and cyclin-dependent kinase 1 (CDK1) were selected for validation of the downregulated subset, and fibronectin 1 (FN1) and glutamine synthetases (GLUL) were chosen as representative candidates of the upregulated subset. Expression levels of FN1 and GLUL were found to be enriched in dT-EVs compared to pT counterparts, thereby corroborating the expression differences identified by mass spectrometry (Figure 6). Both candidate proteins were almost undetectable in protein lysates of parental cells, although mass spectrometry identified GLUL 1.83-fold upregulated in dT- compared to pT-cells but lacking statistical significance (Supplementary Table S1). In contrast, lower levels of CTGF and CDK1 proteins in dT-EVs compared to pT-EVs indicated downregulation, thus confirming the SILAC-based quantification (Figure 6). The protein CTGF was neither detected in cell lysates by mass spectrometry nor by Western blot analysis.

The expression of the EV marker protein TSG101 remained unaffected by the TGFBR2 expression status. Likewise, TGFBR2-regulated protein changes were not observed in EVs isolated from HCT116-AWE17 cells that lack the dox-inducible TGFBR2 expression switch hence excluding any dox-related effect on EV protein levels. In summary, the observed expression differences of at least some TGFBR2-regulated EV proteins have been proven by two different methods, i.e., Western blot analysis and SILAC-based mass spectrometry.

### 2.4. Protein-Protein Network Analysis and Functional Enrichment Analysis of EV Proteins Upregulated by TGFBR2 Deficiency

**Figure 7:**
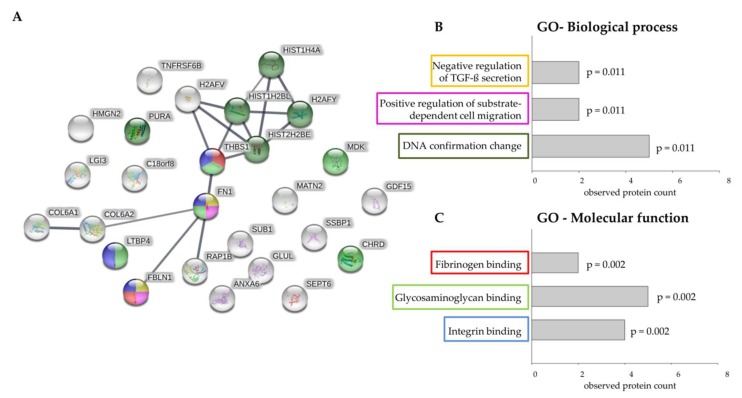
Prediction of protein interactions between upregulated proteins and functional enrichment analysis. (**A**) Protein interaction map of upregulated proteins identified in TGFBR2-deficient EVs (generated by STRING (v11.0)). The connecting lines between protein nodes represent protein-protein interactions. Line thickness indicates the strength of data support (minimum required interaction score = 0.700, high confidence). Coloring of proteins is based on functional enrichment analysis. (**B**) and (**C**) Visible clusters of the protein-protein interaction map were assigned to enriched ontologies for Biological Processes (**B**) and Molecular Function (**C**). Graphs showing most significant enriched Gene Ontology (GO) among the upregulated EV proteins with observed protein count for each category and calculated *p*-value adjusted for multiple comparisons.

Mutational inactivation of *TGFBR2* is considered to be a recurrent driver event in the progression of MSI tumors. In this context, EV proteins that are upregulated by cellular TGFBR2 deficiency are of particular clinical relevance because knowledge about their function and potential interaction partners might provide some clues about their role in MSI tumor biology. Therefore, information on protein–protein interaction (PPI) obtained from STRING database was used to construct a PPI network of 26 EV proteins found to be upregulated by TGFBR2 deficiency (Figure 7A). A calculated PPI enrichment *p*-value of 6.22 × 10^−7^ confirmed statistical significance of detected protein relations. Among the 26 proteins upregulated in dT-EVs, 11 proteins presented high connectivity in form of visible clusters in the PPI network. Subsequent analysis of functional enrichment revealed that “negative regulation of TGF-ß secretion (GO:2001202)” was the most significant affected biological process followed by “positive regulation of substrate-dependent cell migration (GO:1904237)”, and “DNA conformation change (GO:0071103)” (Figure 7B). In the category of molecular function, the upregulated proteins detected in dT-EV appeared to be enriched in “fibrinogen binding (GO:0070051)”, glycosaminoglycan binding (GO:0005539)”, and “integrin binding (GO:0005178)” (Figure 7C). These findings indicate that the functional role of EVs in CRCs is impacted by their protein cargo, which can be altered by a single tumor driver mutation.

## 3. Discussion

Delineation of complex EV cargo changes linked to specific driver mutations is a major challenge due to high heterogeneity of secreted EVs and their parental tumor cells. To overcome this obstacle, we isolated EVs from a TGFBR2-reconsituted MSI CRC model cell line and performed relative protein quantification from dT- and pT-EVs using SILAC labeling. Bottom-up mass spectrometry identified 48 proteins whose expression was altered in dT-EVs but remained unaffected in parental dT-cells. In independent experiments, we successfully validated the differential expression of four of these TGFBR2-regulated proteins (FN1, GLUL, CTGF, CDK1).

On a technical level, we have established a robust protocol for determining EV cargo alteration caused by TGFBR2 deficiency as demonstrated by high reproducibility between the replicates and the successful validation of TGFBR2-regulated proteins in independent EV samples. Although the majority of proteins was found to be shared between EVs and their parental cells, two subsets of EV- and cell-specific proteins were detected. Proteins exclusively quantified in cells might not be sorted into EVs and thus were not identified in EV samples by mass spectrometry. In contrast, we assume that EV-specific proteins were not detected in parental cells due to higher complexity of the cellular proteome [34].

On a biological level, our work emphasizes the capacity of TGFBR2 to alter the protein makeup of EVs and to modulate the molecular message conveyed by these small vesicles. Since the functional role of EVs is determined by their cargo, we hypothesize that altered protein expression levels are biologically relevant and might contribute to the MSI-specific tumor characteristics. In this study, the strongest upregulation was observed for the validated candidate FN1 in dT-EVs. It has been reported that FN1 plays a pro-tumorigenic role in CRC and its downregulation inhibited CRC cell proliferation, migration, and invasion [35,36,37]. Also, it has been shown that TGFBR2 signaling can induce *FN1* expression [38]. This raises the question of how the protein levels of FN1 are increased in dT-EVs as compared to pT-EVs. Although we cannot exclude that TGFBR2 signaling itself might be involved in cargo sorting, other cellular mechanisms might drive the increased sorting of FN1 into dT-EVs. It has been suggested that FN1 is targeted to multivesicular bodies in association with integrins [39], which allows the generation of FN1-expressing EVs (“exosomes”) [40]. We have previously reported that reconstituted TGFBR2 signaling can modulate sialylation of cell surface proteins like ß1-integrin [32]. Whether an interaction between integrins and FN1 might account for the increased FN1 levels detected in dT-EVs warrants further investigation.

Different studies have identified FN1 in EVs secreted from trophoblasts, breast cancer-, melanoma-, neuroblastoma-, and CRC cells [41,42,43,44,45,46]. Chen et al. showed that FN1 is upregulated in serum EVs isolated from CRC patients compared to EVs isolated from serum of healthy controls [46]. In line with this observation, it was reported that FN1 is stronger expressed in intestinal tumor tissues compared to normal colon cells [47]. Moreover, a dense infiltration with lymphocytes was associated with high abundance of FN1 [47], which is a typical histopathological feature observed in most primary MSI CRC tumors [2]. However, evidence for the genomic instability status/mutation profile of CRC patients is lacking in the aforementioned study, making it impossible to correlate increased FN1 expression with the patients’ TGFBR2 status. To the best of our knowledge, our study provides first time evidence for increased FN1 protein expression in dT-EVs.

Apart from expression analysis, functional studies elucidated that FN1 contains several binding domains for integrins, heparins/heparan sulfates, collagens/gelatins, and fibrins [48,49]. It has been demonstrated that FN1 is present on the surface of myeloma cell-derived EVs and can serve as a heparan-binding ligand, which facilitated EV-target cell interactions [43]. FN1-mediated adhesion of EVs to recipients activated intracellular non-canonical TGF-ß signaling and target gene expression, which implicates a fundamental mechanism important for EV-mediated crosstalk [43]. Besides serving as a ligand for members of the glycosaminoglycan family, it has been proposed that EV-associated FN1 can bind to integrin surface receptors on macrophages, which leads to pro-inflammatory IL-1β production [41]. The binding capacity of dT-EVs is supported by our functional enrichment analysis. In addition to FN1, the upregulated candidates THBS1, LTBP4, FBLN1, MDK, and CHRD were associated with fibrinogen-, glycosaminoglycan-, integrin binding. Several histones were also detected to be increased in dT-EVs. There is evidence showing that EV-associated histones have a pro-inflammatory activity and can mediate the endocytosis/uptake of EVs by tumor cells that express syndecan-4 [50,51]. Although not yet tested in experimental studies, we hypothesize that these upregulated proteins in dT-EVs can promote binding/uptake by specific target cells, which is expected to elicit a biological response.

Such biological responses can for example affect the metabolic activity of recipient cells [52]. Adaption of metabolic pathways is a hallmark of cancer cells, which can favor proliferation and synthesis of biomass [53]. EV-mediated metabolic reprogramming can be caused by their bioactive cargo enzymes and metabolites. For example, cancer-associated fibroblast-derived EVs were found to increase glycolysis and glutamine-dependent reductive carboxylation by inhibiting mitochondrial oxidative phosphorylation in cancer cells [52]. Glutamine can be metabolized from nutrients in the small intestine, but most of the glutamine is generated by de-novo synthesis. GLUL (EC 6.3.1.2) is an adenosine triphosphate (ATP)-dependent enzyme that catalyzes the synthesis of glutamine from glutamate and ammonia. Under glutamine starvation, GLUL can provide *de novo* purine availability and cell growth in glioblastoma cells [54,55]. According to our data, the enzyme GLUL was found to be upregulated in dT-EVs and downregulated in pT-EVs. This is in line with studies proposing that TGF-ß can reduce the number of GLUL-positive hepatocytes in mice [56] and inhibit the enzymatic activity in mice astrocytes [57]. For the gastrointestinal tract, it was reported that GLUL activity is moderately low in the large intestine [58]. However, we could confirm by Western blot analysis that GLUL is upregulated in dT-EVs.

Apart from upregulated dT-EV proteins, we have also identified a subset of downregulated candidates in dT-EVs. Cellular TGFBR2 deficiency caused the downregulation of 22 EV proteins, many of them associated with the proteasome. The strongest downregulation was detected for the protein PLAU in dT-EVs, which in turn implies that PLAU was upregulated in pT-EVs. Tumor cells modulate their local environment to gain invasive properties by releasing ECM degrading proteases such as PLAU [59]. PLAU can convert plasminogen to plasmin that has a broad substrate repertoire, including FN1 [60,61]. In our data, FN1 was identified and previously discussed as the top upregulated candidate in dT-EVs. Interestingly, plasmin can activate latent TGF-ß and thus control the bioavailability of the ligand TGF-ß [62]. Besides activating plasmin, PLAU can also modulate a wide range of processes like adhesion, proliferation, and migration by binding to its cell surface receptor uPAR [63]. At the cellular level, it was reported that TGF-ß action can induce PLAU expression in human mammalian epithelial cells [64] and cancer cells [65,66]. Nevertheless, this paper is the first demonstration on TGF-ß-induced PLAU regulation at the vesicular level.

Another known transcriptional target of the TGF-ß pathway is *CTGF* [67,68,69], which we have identified and validated to be downregulated in dT-EVs and thus to be enriched in pT-EVs. The protein CTGF has been studied for its involvement in ECM maintenance and modulation. There is evidence that CTGF serves as a pro-fibrogenic mediator downstream of TGF-ß [69,70]. Also, it has been shown that canonical and non-canonical routes of TGF-β signaling can induce *CTGF* expression depending on cell type and context [69]. In addition, CTGF itself can stimulate the TGF-ß pathway by blocking the Smad7-mediated negative feedback loop [71,72]. It has been proposed that breast cancer cells can upregulate Smad7, which in turn leads to decreased ERK signaling and diminished expression of CTGF [73]. For CRC, it has been suggested that reduced CTGF expression can support the invasive capacity [74], which is known to be a characteristic of MSI CRC tumor cells that often grow as large tumors with local invasive properties [4,75]. At the EV level, it was shown that CTGF can be transferred between hepatic stellate cells [76]. However, the specific functional role of EV-associated CTGF has to be elucidated in future experimental studies. In the current paper, we provide the first association between malfunction of intracellular TGF-ß signaling in MSI CRC cells and downregulated levels of CTGF detected in dT-EVs, which is expected to impact fibrotic matrix modulation and signal transduction.

TGFBR2-mediated TGF-ß signaling has been implicated in cell cycle arrest [22]. One key player in cell cycle regulation is CDK1, which was found to be downregulated in dT-EVs. The specific function of the serine/threonine CDK1 is only known at the cellular level. In interaction with its cyclin partners, CDK1 can phosphorylate target substrates, which leads to cell cycle progression. It has been demonstrated that CDK1 can indirectly interact with TGFBR2 via cyclin B2, which leads to cell cycle arrest in G1/S phase [77]. Even though CDK1 has been previously identified in the cargo of EVs [33,78], its role has not yet been deciphered at the vesicular level.

In conclusion, the current study provides a comprehensive analysis of proteins in MSI CRC-derived EVs. In particular, SILAC labeling combined with mass spectrometry allowed us to identify 48 EV proteins that turned out to be regulated in a TGFBR2-dependent manner. Although TGF-ß signaling and its target genes have been intensively studied at the cellular level, this work provides the first evidence about quantitative differences in EV protein expression caused by TGFBR2 deficiency. It has been reported that dynamics of TGF-ß signaling can be controlled by ESCRT-mediated TGFBR2 endocytosis [79]. The ESCRT machinery plays an essential role in EV biogenesis and protein cargo sorting [80]. However, whether the interaction of the ESCRT machinery and TGFBR2 expression might have a direct impact on the protein cargo sorting of EVs remains to be resolved. Considering that post-translational modifications can impact the biological activity of EV proteins, the phosphorylation status of these candidates should be analyzed before testing specific functions of TGFBR2-regulated EV proteins in the pathogenesis of MSI tumors.

## 4. Materials and Methods

### 4.1. Cell Culture

HCT116-TGFBR2 and HCT116-AWE17 cells were cultured in DMEM-F12 medium (Thermo Fisher Scientific, Waltham, MA USA) supplemented with 10% fetal bovine serum (FBS; Thermo Fisher Scientific), 100 U/mL penicillin and 100 μg/mL streptomycin (Thermo Fisher Scientific) in 5% CO_2_ atmosphere at 37 °C. The generation of the doxycycline (dox)-inducible model system HCT116-TGFBR2 has been described previously [32].

### 4.2. Stable Isotope Labeling with Amino Acids in Cell Culture (SILAC)

HCT116-TGFBR2 cells were cultured in SILAC DMEM medium containing either light (l-[^12^C_6_,^14^N_4_] arginine (Arg0) and l-[^12^C_6_,^14^N_2_] lysine (Lys0)) or heavy (l-[^13^C_6_,^15^N_4_] arginine (+10.0083 Da; Arg10) and l-[^13^C_6_,^15^N_2_] lysine (+8.0142 Da; Lys8)) forms of 0.798 mM arginine and 0.398 mM lysine (SILANTES, Munich, Germany). The medium was supplemented with 10% dialyzed FBS and 2 mM l-glutamine (SILANTES). 200 μg/mL of l-proline (Sigma-Aldrich, Taufkrirchen, Germany) were added to the SILAC culture medium in order to prevent arginine-to-proline conversion [81]. To ensure full incorporation of the SILAC labels, the incorporation rate was determined after 10 days of labeling using a published R script [82] and reached >95%.

### 4.3. Doxycycline Hyclate Treatment

For the SILAC experiment, HCT116-TGFBR2 cells were cultured in the respective SILAC DMEM medium for 14 days. At a confluence of about 80% (28 × 10^6^ cells/flask), the cells were washed twice with phosphate-buffered saline (PBS; Thermo Fisher Scientific) and subsequently cultured for 16 h in minimal volumes (17 mL/T175) of the respective serum-free SILAC medium containing 10 ng/mL TGF-ß1 (Abcam, Cambridge, UK), 2 mM l-glutamine, and either light or heavy forms of arginine and lysine. Heavy-labeled HCT116-TGFBR2 cells were treated with 0.5 μg/mL doxycycline hyclate (dox; Sigma-Aldrich) to induce reconstituted expression of TGFBR2 (heavy state: TGFBR2-proficient, pT). Light-labeled HCT116-TGFBR2 cells were cultivated in the absence of dox and remained TGFBR2-deficient (light state: TGFBR2-deficient, dT). The SILAC experiment was performed in triplicates. For validation experiments, HCT116-TGFBR2 and HCT116-AWE17 cells were also cultured for 16 h in the presence and absence of dox in serum-free DMEM-F12 medium supplemented with TGF-ß1.

### 4.4. EV Preparation

For EV isolation, culture medium was collected and stored on ice until further processing. Each EV replicate was obtained from 5× T175 flasks of HCT116-TGFBR2 or HCT116-AWE17 cells. Cells were also harvested and stored at −20 °C until protein extraction was performed. Cell culture medium was subjected to sequential centrifugations as previously reported [31]. Briefly, floating cells (480× *g*, 4 °C, 10 min), and cellular debris (2000× *g*, 4 °C, 10 min) were removed. Supernatants were passed through a 0.22 μm filter and concentrated (40-fold) to a final volume of 1 mL using 10,000 molecular weight cut-off Vivaspin 20 centrifugal concentrators (Sartorius, Göttingen, Germany) and by centrifugation at 4000× *g* at 4 °C. Total Exosome Isolation Reagent (Thermo Fisher Scientific) was added according to the manufacturer′s instructions. After addition of miniComplete EDTA-free protease inhibitor cocktail (Roche Diagnostics, Mannheim, Germany) and PhosStop phosphatase inhibitor cocktail (Roche Diagnostics), the samples were incubated on a rotating wheel overnight at 4 °C followed by centrifugation (10,000× *g*, 1 h, 4 °C). EV pellets were lysed in RIPA buffer supplemented with miniComplete EDTA-free protease inhibitor and PhosStop phosphatase inhibitor (Roche Diagnostics) for protein extraction [83] or resuspended in particle-free PBS for EV identification.

### 4.5. Transmission Electron Microscopy (TEM)

Five microliters of EV suspensions in particle-free PBS were left to settle onto 100 mesh formvar-coated copper grids (Plano, Wetzlar, Germany), contrasted with 2% aqueous uranyl acetate (negative stain), air-dried and visualized using a JEM-1400 transmission microscope (JEOL, Peabody, MA, USA) equipped with a Tietz 2 K digital camera (TVIPS, Gauting, Germany) at 80 KV.

### 4.6. Nanoparticle Tracking Analysis (NTA)

Size profiles and particle concentrations were assessed by nanoparticle tracking analysis (NTA) using the ZetaView PMX-220 TWIN Laser system with software 8.05.05 SP2 (Particle Metrix, Inning, Germany) according to the manufacturer’s instructions. EV suspensions were diluted 1:3000 (*v/v*) in particle-free PBS and analyzed at 11 different positions. Data acquisition thresholds were set to a shutter of 100, a sensitivity of 90%, and a frame rate of 30 frames per second.

### 4.7. Protein Extraction and Determination of Protein Concentration

Cellular and EV proteins from both TGFBR2 conditions were extracted in RIPA (50 mM Tris-HCl pH 7.4, 150 mM NaCl, 1% Triton X-100, 1% sodium-deoxycholate, 0.1% SDS, 0.1 mM CaCl_2_, 0.01 mM MgCl_2_) buffer [83] supplemented with miniComplete EDTA-free protease inhibitor and PhosStop phosphatase inhibitor. 250 U/mL of benzonase nuclease (Merck, Darmstadt, Germany) were added to the cell lysates to eliminate nucleic acids. EV and cell lysates were incubated at 4 °C on a rotating wheel for 2 h. After centrifugation at 20,000× *g* for 30 min at 4 °C, protein concentration of supernatants was determined using 2-D Quant Kit reagents (GE Healthcare, Uppsala, Sweden) for proteome analysis or by Bradford assay (Bio-Rad, Hercules, USA) for Western blot analysis according to the manufacturer’s instructions.

### 4.8. In-Solution Tryptic Digestion

Protein lysates from corresponding light (dT) and heavy (pT) labeled samples were mixed in a 1:1 ratio based on their protein concentration (cells: 10 μg; EVs: 210 μg). In order to remove EV Isolation Reagent and protease inhibitors, quantitative protein precipitation was performed in a methanol-chloroform-water mixture according to Wessel and Flugge [84]. Precipitated proteins were dissolved in 10 μL (cells) or 135 μL (EVs) 40 mM NH_4_HCO_3_ (Fluka, Steinheim, Germany) and incubated at 25 °C for 1 h in a thermomixer at 600 rpm. Protein disulfide bonds were completely reduced by 2 mM dithiothreitol (DTT; AppliChem, Darmstadt, Germany) in 40 mM NH_4_HCO_3_ at 45 °C for 1 h at 600 rpm. Thiol groups were alkylated by the addition of iodoacetamide (IAA; Sigma, St. Louis, MO, USA) in 40 mM NH_4_HCO_3_ at a final concentration of 5 mM. Samples were incubated in the dark at 25 °C for 30 min at 600 rpm followed by the addition of DTT to a final concentration of 4 mM and incubation at 37 °C for 15 min at 600 rpm. In-solution digestion was performed overnight at 37 °C with 0.1 μg (cells) and 2.1 μg (EVs) trypsin (Promega, Fitchburg, MA, USA). Due to high peptide concentration, additional 1 μg of typsin were added to the EV samples and incubated at 37 °C for 3 h. 5 ug of each EV peptide mixture were transferred into new tubes. Tryptic peptide mixtures were vacuum-dried in a speed-vac and stored at −20 °C until proteome analysis. Newly information added, please confirm.

### 4.9. Nano-LC-ESI-MS/MS

Tryptic EV peptides (1 μg/replicate) were redissolved in 0.1% trifluoroacetic acid/2.5% hexafluoroisopropanol and separated using a nanoAcquity UPLC system (Waters, Eschborn, Germany). Peptides were trapped on a C18 column (180 μm × 20 mm) with a particle size of 5 μm (Waters). Liquid chromatography separation was performed on a BEH130 C18 main-column (100 μm × 100 mm) with a particle size of 1.7 μm (Waters) at a flow rate of 400 nL/min. Each EV sample was fractionated using a 3 h gradient of solvent A (1% acetonitrile, 0.1% formic acid) and solvent B (99.9% acetonitrile, 0.1% formic acid) in the following sequence: From 0% to 4% B in 1 min, from 4% to 25% B in 139 min, from 25% to 40% B in 15 min, from 40% to 85% B in 10 min, 5 min at 85% B, from 85% to 4% B in 2 min, and 15 min at 4% B. The nanoUPLC system was coupled online to an LTQ Orbitrap XL mass spectrometer (Thermo Fisher Scientific) using a nanoESI interface. Following parameters were set: ESI voltage 2000 V; capillary temperature 200 °C, normalized collision energy 35 V. Data acquisition was performed with Xcalibur software by scan cycles of one Fourier transform mass spectrometry (FTMS) scan with a resolution of 60,000 at m/z 400 and a range from 300 to 2000 m/z in parallel with six MS/MS scans in the ion trap of the most abundant precursor ions.

Cellular peptides mixtures (1 μg/replicate) were separated using the Dionex UltiMate 3000 nanoUPLC system (Thermo Scientific, Bremen, Germany). Peptides were trapped on an Acclaim Pepmap 100 column, 100 μm × 20 mm, particle size 5 μm (Thermo Scientific). The liquid chromatography separation was performed on a C18 column (Acclaim Pepmap RSLC, 75 μm × 50 cm, particle size 2 μm (Thermo Scientific) with a flow rate of 300 nL/min. Chromatography was carried out using a 2 h gradient of solvent C (99.9% water, 0.1% formic acid) and solvent D (80% acetonitrile, 19.9% water, 0.1% formic acid) in the following sequence: 2 min at 2% D, from 2% to 8% D in 1 min, from 8% to 25% D in 80 min, from 25% to 40% D in 10 min, from 40% to 95% D in 1 min, 5 min at 95% D, from 95 to 2% D in 1 min, and 20 min at 2% D. The nanoUPLC system was coupled online to a Q Exactive HF-X Hybrid Quadrupole-Orbitrap mass spectrometer (Thermo Scientific). Following parameters were set: ESI voltage 2200 V; capillary temperature 275 °C, normalized collision energy 35 V. Data were acquired by scan cycles of one FTMS scan with a resolution of 120,000 at m/z 200 and a range from 300 to 2000 m/z in parallel with ten MS/MS scans in the ion trap of the most abundant precursor ions.

The mass spectrometry proteomics raw files have been deposited to the ProteomeXchange Consortium [85] (http://proteomecentral.proteomexchange.org) via the PRIDE partner repository [86] with the dataset identifier PXD013980.

### 4.10. Protein Identification and Quantification

LC-MS/MS raw data was imported to MaxQuant software (version 1.6.3.3) [87]. Peptides were identified using Andromeda search engine [88] against the SwissProt database (download: 01/03/2019; Homo sapiens: 20,412 entries). Enzyme specificity was set to trypsin/p and two missed cleavage sites in case of incomplete trypsin hydrolysis were permitted. A minimum peptide length of 7 amino acids was required. Cysteine carbamidomethylation (C) was set as fixed modification, whereas methionine oxidation (M), asparagine and glutamine deamidation (NQ), and protein *N*-terminal acetylation were considered as variable modifications. According to the SILAC labeling, no labeling or double labeling (Arg10 and Lys8) were selected with maximum of 3 labeled amino acids per peptide. Mass tolerances were defined for precursor and fragmented ions as follows: MS first search-20 ppm, MS main search-6 ppm, and MS/MS-0.5 Da. Identification was performed under a false discovery rate (FDR) of 0.01. SILAC-based quantification was based on unique and razor peptides with a minimum of two ratio counts. Peptide ratios were calculated and normalized for each arginine- and/or lysine-containing peptide as described [89].

### 4.11. Data Analysis

Data analysis was performed using Perseus (version 1.6.1.3) [90,91] and R software (version 3.5.0) with the following packages: forcats_0.4.0, stringr_1.4.0, dplyr_0.8.0.1, purrr_0.3.2, readr_1.3.1, tidyr_0.8.3, tibble_2.1.1, tidyverse_1.2.1, ggrepel_0.8.0, ggplot2_3.1.0, and readxl_1.3.1. Proteins derived from decoy database containing reversed protein sequences, common contaminants (EVs: KRT1, KRT8, KRT19, DMKN, TPM4, mouse KRT18, bovine GSN; cells: KRT1, KRT8, KRT10, KRT19, KRT82, GSN, MT1X) as well as proteins identified by site modification only were strictly excluded from further analysis. Proteins identified with at least two unique peptides and quantified in at least 2 out of 3 biological replicates were considered for further analysis. To define significantly regulated proteins between TGFBR2-proficient (pT; heavy state) and TGFBR2-deficient (dT; light state) conditions, fold changes (dT/pT ratios) were calculated and log_2_ transformation was performed. In order to identify significant TGFBR2-regulated proteins, one-sample t tests were conducted with null hypothesis assuming that the difference in protein ratios between replicates equals 0. Obtained *p*-values were adjusted for multiple testing according to Benjamini and Hochberg [92]. After applying an adjusted *p*-value of ≤ 0.05 as a cut-off, proteins were only classified as significantly regulated if their abundance changed more than 1.5- or less than 0.667-fold (log2 ≥ |0.585|) between the TGFBR2 conditions. Enrichment analysis of regulated proteins was performed in STRING [93] (version 11.0) for Gene Ontology Biological Processes (GO BP) and Gene Ontology Molecular Functions (GO MF).

### 4.12. SDS Page and Western Blot

Protein extracts (40 μg/sample) were separated on 4–20% Bis-Tris gradient gels (Expedeon, San Diego, CA, USA) and blotted onto a nitrocellulose membrane (Life Technologies, Carlsbad, CA, USA). After blocking membranes in 5% milk/TBST, the following primary antibodies were used: Mouse anti-CD63 (1:800, clone MX-49.129.5, Santa Cruz, Heidelberg, Germany), mouse anti-CD9 (1:100, clone C-4, Santa Cruz), mouse anti-CD81 (1:1000, clone TS81, Abcam), mouse anti-β-Actin (1:2000, clone C4, MP Biomedicals, Eschwege, Germany), goat anti-Alix (1:800, sc-49268, Santa Cruz), mouse anti-TSG101 (1:500, clone 4A10, Thermo Fisher Scientific), mouse anti-CNX (1:500, clone E-10, Santa Cruz), mouse anti-FN1 (1:1000, clone A-11, Santa Cruz), rabbit anti-CTGF (1:1000; #86641, Cell Signaling), mouse anti-GLUL (1:100, clone E-4, Santa Cruz), rabbit anti-CDK1 (1:1000, #77055, Cell Signaling), mouse anti-TGFBR2 (1:300, clone D2, Santa Cruz), rabbit anti-Smad2 (1:1000, clone 86F7, Cell Signaling), and rabbit anti-pSmad2 (1:1000, Ser465/467, Cell Signaling). Diluted primary antibodies were incubated with membranes overnight at 4 °C. The blots were washed with TBST and incubated with a sheep anti-mouse-IgG HRP (1:5000; GE Healthcare), goat anti-rabbit-IgG HRP (1:2500, Promega) or donkey anti-goat-IgG HRP (1:1000, Santa Cruz) secondary antibody for 1 h at room temperature (RT). Signals were detected using Western Lightning Plus ECL (Perkin Elmer, Waltham, MA, USA) and a ChemiDoc MP System (Bio-Rad).

## Figures and Tables

**Table 1:** 26 Significantly upregulated proteins in dT-EVs.

Gene Name	Protein Name	Protein Ratio ^1^ (dT/pT)	T Test *p*-Value (adj.)	Peptides (unique)	Coverage (%)	Identification Score
*FN1*	Fibronectin	4.73	0.006	31 (31)	19.7	109.14
*HIST1H4A*	Histone H4	2.48	7.79 × 10^-5^	8 (8)	53.4	54.26
*HIST1H2BL*	Histone H2B type 1-L	2.13	2.33 × 10^-4^	6 (2)	41.3	72.66
*HIST2H2BE*	Histone H2B type 2-E	2.03	3.25 × 10^-4^	6 (2)	41.3	4.33
*H2AFV*	Histone H2A.V	1.98	0.006	5 (3)	53.9	44.88
*GLUL*	Glutamine synthetase	1.77	0.010	6 (6)	20.1	24.52
*C18orf8*	Uncharacterized protein C18orf8	1.74	0.018	2 (2)	4.4	2.61
*ANXA6*	Annexin A6	1.68	0.002	12 (12)	22.3	23.43
*FBLN1*	Fibulin-1	1.64	0.018	26 (13)	51.9	323.21
*SUB1*	Activated RNA polymerase II transcriptional coactivator p15	1.64	0.001	6 (6)	33.9	21.71
*RAP1B*	Ras-related protein Rap-1b	1.62	0.008	5 (5)	35.9	17.40
*LTBP4*	Latent-transforming growth factor beta-binding protein 4	1.61	3.27 × 10^-5^	32 (32)	30.8	302.21
*MDK*	Midkine	1.58	7.74 × 10^-6^	5 (5)	32.9	138.01
*SSBP1*	Single-stranded DNA-binding protein, mitochondrial	1.57	0.004	4 (4)	29.1	8.99
*COL6A1*	Collagen alpha-1(VI) chain	1.57	4.37 × 10^-6^	20 (20)	26.5	323.31
*THBS1*	Thrombospondin-1	1.57	6.54 × 10^-5^	50 (14)	56.8	323.31
*CHRD*	Chordin	1.57	0.002	7 (7)	12.5	14.76
*SEPT6*	Septin-6	1.56	0.007	5 (2)	20.0	5.35
*TNFRSF6B*	Tumor necrosis factor receptor superfamily member 6B	1.55	0.001	3 (3)	16.0	17.80
*LGI3*	Leucine-rich repeat LGI family member 3	1.55	0.002	10 (10)	25.0	39.49
*PURA*	Transcriptional activator protein Pur-alpha	1.54	0.003	3 (3)	17.7	6.24
*COL6A2*	Collagen alpha-2(VI) chain	1.54	0.001	12 (12)	18.4	58.43
*GDF15*	Growth/differentiation factor 15	1.53	0.001	20 (20)	65.3	127.44
*HMGN2*	Non-histone chromosomal protein HMG-17	1.53	3.42 × 10^-4^	3 (3)	16.7	6.75
*H2AFY*	Core histone macro-H2A.1	1.51	0.006	8 (8)	34.4	26.84
*MATN2*	Matrilin-2	1.50	0.001	23 (23)	31.2	190.93

^1^ Mean ratios calculated from three biological replicates.

**Table 2:** 22 Significantly downregulated proteins in dT-EVs.

Gene Name	Protein Name	Protein Ratio ^1^ (dT/pT)	T Test *p*-Value (adj.)	Peptides (unique)	Coverage (%)	Identification Score
*PLAU*	Urokinase-type plasminogen activator	0.46	1.73 × 10^-4^	16 (16)	50.6	323.31
*CTGF*	Connective tissue growth factor	0.51	2.95 × 10^-4^	15 (15)	55.3	66.74
*PSMB1*	Proteasome subunit beta type-1	0.55	0.007	5 (5)	29	12.98
*PSMB5*	Proteasome subunit beta type-5	0.55	0.001	5 (5)	25.5	20.07
*PSMA2*	Proteasome subunit alpha type-2	0.58	0.010	4 (4)	21.4	20.44
*MCM7*	DNA replication licensing factor MCM7	0.59	1.51 × 10^-5^	12 (12)	22.4	34.56
*SAFB*	Scaffold attachment factor B1	0.60	0.023	6 (6)	14.6	46.12
*PSMB2*	Proteasome subunit beta type-2	0.61	0.002	3 (3)	17.9	27.04
*PSMB4*	Proteasome subunit beta type-4	0.61	0.007	3 (3)	14.8	4.97
*CCAR2*	Cell cycle and apoptosis regulator protein 2	0.62	0.002	8 (8)	13.2	22.57
*TARDBP*	TAR DNA-binding protein 43	0.63	0.006	2 (2)	7.2	5.87
*APRT*	Adenine phosphoribosyltransferase	0.64	0.004	5 (5)	37.2	43.68
*PSMB3*	Proteasome subunit beta type-3	0.64	2.35 × 10^-4^	4 (4)	30.7	14.65
*ALDH1A3*	Aldehyde dehydrogenase family 1 member A3	0.65	0.001	12 (12)	28.5	57.51
*PSMA1*	Proteasome subunit alpha type-1	0.65	0.001	8 (8)	36.9	14.21
*UHRF1*	E3 ubiquitin-protein ligase UHRF1	0.65	0.006	8 (8)	13.7	9.60
*CDK1*	Cyclin-dependent kinase 1	0.65	0.006	9 (8)	39.4	22.58
*PSMA3*	Proteasome subunit alpha type-3	0.65	0.002	8 (8)	34.1	23.82
*MCM6*	DNA replication licensing factor MCM6	0.66	0.002	16 (16)	26.4	43.74
*HSPD1*	60 kDa heat shock protein, mitochondrial	0.66	0.001	14 (14)	34.0	108.37
*CTBP2*	C-terminal-binding protein 2	0.66	0.012	5 (4)	18.2	16.52
*PSMA5*	Proteasome subunit alpha type-5	0.66	0.001	7 (7)	40.2	34.82

^1^ Mean ratios calculated from three biological replicates.

## Supplementary Materials

Supplementary Materials can be found at https://www.mdpi.com/1422-0067/20/17/4162/s1.

## Abbreviations

CRC	Colorectal cancer
Dox	Doxycycline
dT	Transforming Growth Factor Beta Receptor Type 2-deficient
ECM	Extracellular matrix
EVs	Extracellular vesicles
FTMS	Fourier transform mass spectrometry
MISEV	Minimal information for studies of extracellular vesicles
MSI	Microsatellite instability
PPI	Protein-protein interaction
pT	Transforming Growth Factor Beta Receptor Type 2-proficient
SILAC	Stable isotope labeling with amino acids in cell culture
TGFBR2	Transforming Growth Factor Beta Receptor Type 2
