# Supplementary material for: SILAC-Based Quantification of TGFBR2-Regulated Protein Expression in Extracellular Vesicles of Microsatellite Unstable Colorectal Cancers
Source: Int J Mol Sci. 2019 Aug 26;20(17):4162. doi: 10.3390/ijms20174162 (PMC6747473; doi:10.3390/ijms20174162)
Supplement: Supplementary file 1 [file ijms-20-04162-s001.pdf]

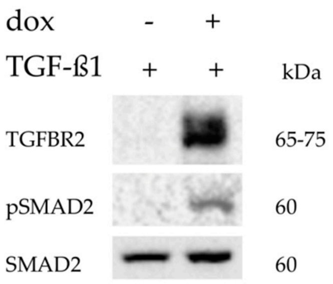


**Supplementary Figure S1.** Doxycycline-induced reconstitution of TGFBR2 expression and downstream signaling. Western blot analysis demonstrated dox-induced reconstituted expression of TGFBR2 and phosphorylation of SMAD2 (pSMAD2) in protein lysates of HCT116-TGFBR2 cells. Total SMAD2 protein was used as a loading control. Protein sizes are indicated. .

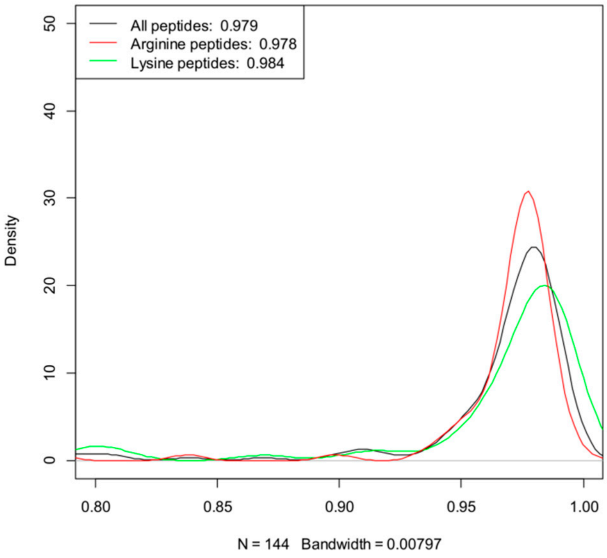


**Supplementary Figure S2** Incorporation of heavy-labeled amino acids in EVs isolated from TGFBR2-proficient HCT116-TGFBR2 cells. Incorporation efficiency of heavy-labeled amino acids was calculated based on 144 identified peptides.

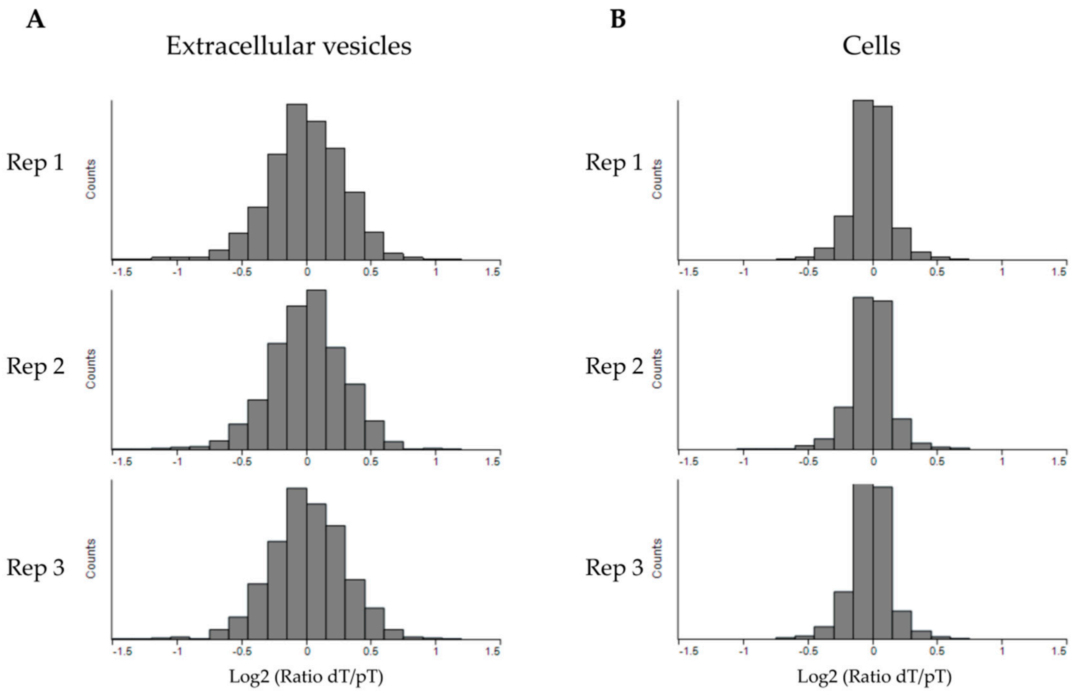


**Supplementary Figure S3.** Distribution of quantified protein ratios. Histograms are shown for $log_2$-transformed protein ratios (dT/pT) from each biological replicate of EVs (A) and cells (B).

**Supplementary Table S1** TGFBR2-regulated proteins in cells.

| Gene names | Protein names | Protein ratio [1] (dT/pT) | Peptides (unique) | Coverage (%) | Identification score |
|---|---|---|---|---|---|
| *Upregulated cellular proteins by TGFBR2 deficiency* | | | | | |
| NUMA1 | Nuclear mitotic apparatus protein 1 | 2.23 | 12 (12) | 8.3 | 36.38 |
| CAD | CAD protein | 1.91 | 20 (20) | 13.1 | 85.112 |
| GLUL | Glutamine synthetase | 1.82 | 7 (7) | 25.5 | 29.43 |
| UBE2Z | Ubiquitin-conjugating enzyme E2 Z | 1.77 | 4 (4) | 13.3 | 13.23 |
| TUBB4A | Tubulin beta-4A chain | 1.71 | 17 (17) | 6.3 | 26.76 |
| EDC4 | Enhancer of mRNA-decapping protein 4 | 1.56 | 7 (7) | 9.0 | 27.22 |
| RANBP2 | E3 SUMO-protein ligase RanBP2 | 1.51 | 6 (6) | 3.4 | 20.66 |
| *Downregulated cellular proteins by TGFBR2 deficiency* | | | | | |
| MGST3 | Microsomal glutathione S-transferase 3 | 0.61 | 3 (3) | 32.2 | 15.32 |

| | | | | | |
|---|---|---|---|---|---|
| KRT18 | Keratin, type I cytoskeletal 18 | 0.61 | 30 (19) | 77 | 323.31 |
| VDAC3 | Voltage-dependent anion-selective channel protein 3 | 0.63 | 9 (9) | 41.7 | 63.09 |
| EMG1 | Ribosomal RNA small subunit methyltransferase NEP1 | 0.64 | 4 (4) | 22.1 | 15.02 |
| KRT8 | Keratin, type II cytoskeletal 8 | 0.65 | 39 (31) | 64.2 | 323.31 |
| VDAC2 | Voltage-dependent anion-selective channel protein 2 | 0.66 | 13 (13) | 59.5 | 174.22 |
| HN1 | Hematological and neurological expressed 1 protein | 0.67 | 3 (3) | 54.5 | 18.21 |

[1] Mean ratios calculated from three biological replicates.
